# *N*-{[4-(4-Meth­oxy­benzene­sulfonamido)­phen­yl]sulfon­yl}acetamide

**DOI:** 10.1107/S1600536810023925

**Published:** 2010-06-23

**Authors:** Ghulam Mustafa, Mehmet Akkurt, Islam Ullah Khan, Rahat Naseem, Beenish Sajjad

**Affiliations:** a Department of Chemistry, University of Gujrat, H. H. Campus, Gujrat 50700, Pakistan; b Department of Physics, Faculty of Arts and Sciences, Erciyes University, 38039 Kayseri, Turkey; c Materials Chemistry Laboratory, Department of Chemistry, Government College University, Lahore 54000, Pakistan

## Abstract

In the title compound, C_15_H_16_N_2_O_6_S_2_, the dihedral angle between the benzene rings is 83.2 (3)°. The mol­ecular conformation is stabilized by an intra­molecular C—H⋯O inter­action. In the crystal structure, mol­ecules are linked by N—H⋯O and C—H⋯O hydrogen bonds and additional stabilization is provided by weak C—H⋯π inter­actions.

## Related literature

For previous studies on the synthesis of sulfonamide derivatives with phenyl glycine, see: Ashfaq *et al.* (2009 ▶, 2010 ▶).
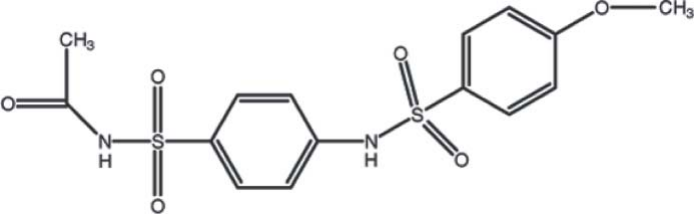


## Experimental

### 

#### Crystal data

C_15_H_16_N_2_O_6_S_2_
*M* *_r_* = 384.44
Monoclinic, 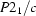

*a* = 5.3651 (10) Å
*b* = 20.551 (3) Å
*c* = 15.034 (2) Å
β = 94.040 (7)°
*V* = 1653.5 (4) Å^3^
*Z* = 4
Mo *K*α radiation
μ = 0.36 mm^−1^
*T* = 296 K
0.25 × 0.08 × 0.07 mm

#### Data collection

Bruker Kappa APEXII CCD diffractometer
13678 measured reflections
3771 independent reflections
1608 reflections with *I* > 2σ(*I*)
*R*_int_ = 0.114

#### Refinement

*R*[*F* ^2^ > 2σ(*F* ^2^)] = 0.084
*wR*(*F* ^2^) = 0.189
*S* = 0.89
3771 reflections
226 parameters
H-atom parameters constrained
Δρ_max_ = 0.83 e Å^−3^
Δρ_min_ = −0.32 e Å^−3^

### 

Data collection: *APEX2* (Bruker, 2007 ▶); cell refinement: *SAINT* (Bruker, 2007 ▶); data reduction: *SAINT*; program(s) used to solve structure: *SHELXS97* (Sheldrick, 2008 ▶); program(s) used to refine structure: *SHELXL97* (Sheldrick, 2008 ▶); molecular graphics: *ORTEP-3* (Farrugia, 1997 ▶); software used to prepare material for publication: *WinGX* (Farrugia, 1999 ▶) and *PLATON* (Spek, 2009 ▶).

## Supplementary Material

Crystal structure: contains datablocks global, I. DOI: 10.1107/S1600536810023925/hb5502sup1.cif

Structure factors: contains datablocks I. DOI: 10.1107/S1600536810023925/hb5502Isup2.hkl

Additional supplementary materials:  crystallographic information; 3D view; checkCIF report

## Figures and Tables

**Table 1:** Hydrogen-bond geometry (Å, °) *Cg*1 and *Cg*2 are the centroids of the C1–C6 and C8–C13 rings, respectively.

*D*—H⋯*A*	*D*—H	H⋯*A*	*D*⋯*A*	*D*—H⋯*A*
N1—H1⋯O4^i^	0.86	2.09	2.932 (5)	168
N2—H2⋯O5^ii^	0.86	2.26	3.071 (5)	157
C13—H13⋯O2	0.93	2.35	2.986 (6)	126
C15—H15*C*⋯O6^ii^	0.96	2.45	3.348 (7)	156
C15—H15*B*⋯*Cg*1^iii^	0.96	2.79	3.722 (6)	164
C15—H15*A*⋯*Cg*2^iii^	0.96	2.79	3.589 (6)	141

Symmetry codes: (i) 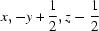; (ii) 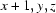; (iii) 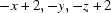.

**Fig. 1.**
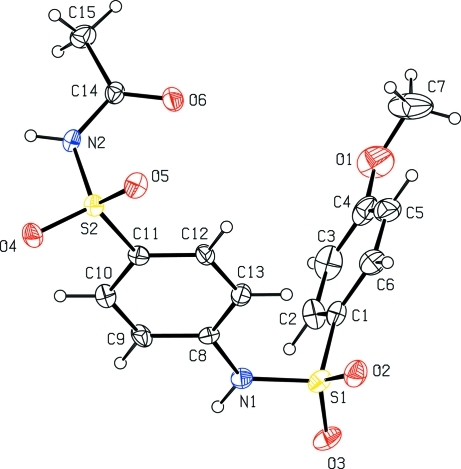
The title molecule with displacement ellipsoids for non-H atoms drawn at the 30% probability level.

**Fig. 2.**
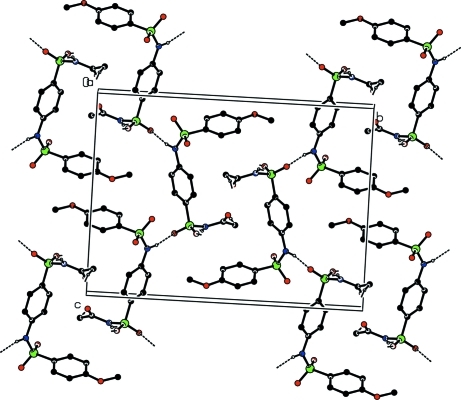
The packing and hydrogen bonding of (I) viewed down a axis. H atoms not participating in hydrogen bonding have been omitted for clarity.

## supplementary crystallographic information

### Comment

Sulphacetamide sodium is an antibiotic which is being used for eye infections. Because the antiobiotics lose their efficacy after long term used, so there is a need to derivatize them to get better therapeutic result. In this paper, a new derivative of it is being reported. Previously this drug has also been derivatized by other researchers (Ashfaq *et al.*, 2009, 2010). Here we present the crystal structure of the title compound (I), (Fig. 1).

In (I), the benzene rings (C1–C6) and (C8–C13) are twisted with a dihedral angle of 83.2 (3) ° to each other. Molecular conformation is stabilized by intramolecular C–H···O interactions. Intermolecular N—H···O and C—H···O hydrogen bonds and C—H···π interactions contribute to the stabilization of the crystal structure (Table 1, Fig. 2).

### Experimental

Sodium sulphacetamide (0.5 g, 2.32 mmol) was taken in 50 ml round bottom flask and dissolved in 20 ml of distilled water. Then, methoxy benzene sulfonyl chloride (0.46 g, 2.32 mmol) was added with continuous stirring at ambient temperature. The pH of this solution was strictly maintained between 8 and 9 by using NaHCO_3_ (3 *M*). The consumption of suspended methoxy benzene sulfonyl chloride was an indication of reaction completion. Then pH was adjusted to 2–3 using HCl (3 N). The precipitates formed were filtered, washed three to four times with distilled water and recrystallised using methanol to yield colourless rods of (I).

### Refinement

All H-atoms were positioned geometrically and refined using a riding model with d(N—H) = 0.86 Å, *U*_iso_ = 1.2*U*_eq_(N) for NH, 0.93 Å, *U*_iso_ = 1.2*U*_eq_(C) for aromatic and 0.96 Å, *U*_iso_ = 1.5*U*_eq_(C) for CH_3_ hydrogen atoms.

### Crystal data

**Table:**

C_15_H_16_N_2_O_6_S_2_	*F*(000) = 800
*M**_r_* = 384.44	*D*_x_ = 1.544 Mg m^−^^3^
Monoclinic, *P*2_1_/*c*	Mo *K*α radiation, λ = 0.71073 Å
Hall symbol: -P 2ybc	Cell parameters from 1463 reflections
*a* = 5.3651 (10) Å	θ = 2.9–20.1°
*b* = 20.551 (3) Å	µ = 0.36 mm^−^^1^
*c* = 15.034 (2) Å	*T* = 296 K
β = 94.040 (7)°	Rod, colourless
*V* = 1653.5 (4) Å^3^	0.25 × 0.08 × 0.07 mm
*Z* = 4	

### Data collection

**Table:**

Bruker Kappa APEXII CCD diffractometer	1608 reflections with *I* > 2σ(*I*)
Radiation source: sealed tube	*R*_int_ = 0.114
graphite	θ_max_ = 28.4°, θ_min_ = 3.3°
phi and ω scans	*h* = −7→7
13678 measured reflections	*k* = −27→26
3771 independent reflections	*l* = −20→20

### Refinement

**Table:**

Refinement on *F*^2^	Primary atom site location: structure-invariant direct methods
Least-squares matrix: full	Secondary atom site location: difference Fourier map
*R*[*F*^2^ > 2σ(*F*^2^)] = 0.084	Hydrogen site location: inferred from neighbouring sites
*wR*(*F*^2^) = 0.189	H-atom parameters constrained
*S* = 0.89	*w* = 1/[σ^2^(*F*_o_^2^) + (0.068*P*)^2^] where *P* = (*F*_o_^2^ + 2*F*_c_^2^)/3
3771 reflections	(Δ/σ)_max_ < 0.001
226 parameters	Δρ_max_ = 0.83 e Å^−^^3^
0 restraints	Δρ_min_ = −0.32 e Å^−^^3^

### Special details

**Table:**

Geometry. Bond distances, angles *etc*. have been calculated using the rounded fractional coordinates. All su's are estimated from the variances of the (full) variance-covariance matrix. The cell e.s.d.'s are taken into account in the estimation of distances, angles and torsion angles
Refinement. Refinement on *F*^2^ for ALL reflections except those flagged by the user for potential systematic errors. Weighted *R*-factors *wR* and all goodnesses of fit *S* are based on *F*^2^, conventional *R*-factors *R* are based on *F*, with *F* set to zero for negative *F*^2^. The observed criterion of *F*^2^ > σ(*F*^2^) is used only for calculating -*R*-factor-obs *etc*. and is not relevant to the choice of reflections for refinement. *R*-factors based on *F*^2^ are statistically about twice as large as those based on *F*, and *R*-factors based on ALL data will be even larger.

### Fractional atomic coordinates and isotropic or equivalent isotropic displacement parameters (Å^2^)

**Table:**

	*x*	*y*	*z*	*U*_iso_*/*U*_eq_	
S1	0.3465 (3)	0.17871 (7)	0.68802 (9)	0.0373 (5)	
S2	0.7590 (2)	0.14262 (6)	1.14309 (8)	0.0290 (4)	
O1	0.7672 (11)	−0.0692 (3)	0.5765 (3)	0.082 (2)	
O2	0.1176 (7)	0.16711 (19)	0.7264 (2)	0.0455 (14)	
O3	0.3551 (7)	0.2208 (2)	0.6128 (2)	0.0515 (16)	
O4	0.8828 (6)	0.19627 (17)	1.1877 (2)	0.0386 (11)	
O5	0.5388 (6)	0.11632 (18)	1.1764 (2)	0.0377 (11)	
O6	0.7458 (7)	0.00642 (18)	1.0809 (3)	0.0444 (14)	
N1	0.5446 (8)	0.2099 (2)	0.7634 (2)	0.0348 (14)	
N2	0.9779 (7)	0.0863 (2)	1.1487 (3)	0.0335 (14)	
C1	0.4677 (10)	0.1033 (3)	0.6585 (3)	0.0373 (19)	
C2	0.6748 (11)	0.1020 (3)	0.6078 (4)	0.051 (2)	
C3	0.7658 (13)	0.0438 (4)	0.5824 (4)	0.060 (3)	
C4	0.6569 (12)	−0.0140 (4)	0.6055 (4)	0.055 (2)	
C5	0.4498 (13)	−0.0129 (3)	0.6561 (4)	0.054 (2)	
C6	0.3601 (11)	0.0463 (3)	0.6810 (4)	0.0450 (19)	
C7	0.671 (2)	−0.1301 (4)	0.5999 (5)	0.102 (4)	
C8	0.5896 (9)	0.1915 (2)	0.8531 (3)	0.0274 (16)	
C9	0.7968 (9)	0.2181 (3)	0.8989 (3)	0.0345 (17)	
C10	0.8521 (9)	0.2034 (3)	0.9879 (3)	0.0353 (17)	
C11	0.6938 (9)	0.1611 (2)	1.0304 (3)	0.0278 (16)	
C12	0.4918 (9)	0.1342 (2)	0.9844 (3)	0.0322 (17)	
C13	0.4361 (10)	0.1492 (3)	0.8956 (3)	0.0345 (17)	
C14	0.9434 (9)	0.0225 (3)	1.1177 (3)	0.0310 (17)	
C15	1.1613 (10)	−0.0206 (3)	1.1364 (4)	0.0426 (17)	
H1	0.63300	0.24180	0.74610	0.0420*	
H2	1.12300	0.09690	1.17230	0.0400*	
H2A	0.75010	0.14060	0.59160	0.0610*	
H3	0.90470	0.04290	0.54880	0.0720*	
H5	0.37410	−0.05130	0.67260	0.0650*	
H6	0.22080	0.04750	0.71450	0.0540*	
H7A	0.66710	−0.13270	0.66350	0.1520*	
H7B	0.77580	−0.16400	0.57940	0.1520*	
H7C	0.50490	−0.13500	0.57270	0.1520*	
H9	0.90000	0.24620	0.86980	0.0420*	
H10	0.99190	0.22120	1.01900	0.0420*	
H12	0.39030	0.10540	1.01310	0.0380*	
H13	0.29670	0.13110	0.86470	0.0420*	
H15A	1.12360	−0.06320	1.11290	0.0640*	
H15B	1.19890	−0.02350	1.19970	0.0640*	
H15C	1.30280	−0.00320	1.10880	0.0640*	

### Atomic displacement parameters (Å^2^)

**Table:**

	*U*^11^	*U*^22^	*U*^33^	*U*^12^	*U*^13^	*U*^23^
S1	0.0361 (8)	0.0443 (9)	0.0310 (7)	0.0051 (6)	−0.0015 (6)	0.0047 (6)
S2	0.0303 (7)	0.0313 (7)	0.0256 (6)	0.0004 (5)	0.0039 (5)	−0.0046 (5)
O1	0.110 (4)	0.070 (4)	0.066 (3)	0.033 (3)	0.003 (3)	−0.024 (3)
O2	0.032 (2)	0.054 (3)	0.050 (2)	0.0047 (18)	−0.0006 (17)	−0.0042 (19)
O3	0.061 (3)	0.058 (3)	0.034 (2)	0.008 (2)	−0.0071 (19)	0.0161 (19)
O4	0.045 (2)	0.034 (2)	0.0362 (19)	−0.0048 (17)	−0.0013 (17)	−0.0108 (17)
O5	0.0298 (19)	0.050 (2)	0.0343 (19)	0.0003 (17)	0.0090 (15)	−0.0001 (17)
O6	0.034 (2)	0.035 (2)	0.063 (3)	−0.0026 (17)	−0.0055 (18)	−0.0050 (19)
N1	0.040 (2)	0.036 (3)	0.028 (2)	−0.006 (2)	−0.0013 (19)	0.0099 (19)
N2	0.027 (2)	0.038 (3)	0.034 (2)	−0.0021 (19)	−0.0081 (18)	0.001 (2)
C1	0.033 (3)	0.048 (4)	0.030 (3)	−0.001 (3)	−0.005 (2)	−0.002 (2)
C2	0.046 (4)	0.056 (4)	0.050 (4)	−0.010 (3)	0.003 (3)	−0.011 (3)
C3	0.050 (4)	0.083 (5)	0.048 (4)	0.006 (4)	0.010 (3)	−0.018 (4)
C4	0.054 (4)	0.067 (5)	0.042 (3)	0.019 (4)	−0.014 (3)	−0.015 (3)
C5	0.072 (5)	0.045 (4)	0.043 (3)	0.007 (3)	−0.010 (3)	0.002 (3)
C6	0.048 (3)	0.052 (4)	0.035 (3)	0.003 (3)	0.004 (3)	0.003 (3)
C7	0.189 (10)	0.054 (5)	0.060 (5)	0.039 (6)	−0.002 (6)	−0.005 (4)
C8	0.029 (3)	0.025 (3)	0.028 (2)	0.004 (2)	0.001 (2)	0.005 (2)
C9	0.036 (3)	0.033 (3)	0.035 (3)	−0.014 (2)	0.007 (2)	0.005 (2)
C10	0.033 (3)	0.036 (3)	0.037 (3)	−0.004 (2)	0.003 (2)	−0.004 (2)
C11	0.033 (3)	0.026 (3)	0.025 (2)	0.002 (2)	0.006 (2)	−0.002 (2)
C12	0.034 (3)	0.029 (3)	0.033 (3)	−0.009 (2)	−0.001 (2)	0.003 (2)
C13	0.031 (3)	0.039 (3)	0.033 (3)	−0.004 (2)	−0.002 (2)	0.004 (2)
C14	0.026 (3)	0.037 (3)	0.030 (3)	−0.004 (2)	0.002 (2)	0.003 (2)
C15	0.037 (3)	0.041 (3)	0.050 (3)	0.013 (3)	0.004 (2)	−0.002 (3)

### Geometric parameters (Å, °)

**Table:**

S1—O2	1.413 (4)	C8—C13	1.384 (7)
S1—O3	1.427 (4)	C8—C9	1.379 (7)
S1—N1	1.629 (4)	C9—C10	1.383 (7)
S1—C1	1.750 (6)	C10—C11	1.401 (7)
S2—O4	1.430 (4)	C11—C12	1.361 (7)
S2—O5	1.422 (3)	C12—C13	1.382 (6)
S2—N2	1.647 (4)	C14—C15	1.478 (8)
S2—C11	1.747 (5)	C2—H2A	0.9300
O1—C4	1.365 (10)	C3—H3	0.9300
O1—C7	1.408 (10)	C5—H5	0.9300
O6—C14	1.207 (6)	C6—H6	0.9300
N1—C8	1.405 (5)	C7—H7A	0.9600
N2—C14	1.399 (7)	C7—H7B	0.9600
N1—H1	0.8600	C7—H7C	0.9600
N2—H2	0.8600	C9—H9	0.9300
C1—C6	1.359 (9)	C10—H10	0.9300
C1—C2	1.391 (8)	C12—H12	0.9300
C2—C3	1.357 (10)	C13—H13	0.9300
C3—C4	1.379 (11)	C15—H15A	0.9600
C4—C5	1.390 (9)	C15—H15B	0.9600
C5—C6	1.370 (9)	C15—H15C	0.9600
			
O2—S1—O3	120.2 (2)	S2—C11—C12	120.1 (3)
O2—S1—N1	109.0 (2)	C10—C11—C12	120.5 (4)
O2—S1—C1	107.7 (3)	C11—C12—C13	120.7 (4)
O3—S1—N1	104.9 (2)	C8—C13—C12	119.4 (5)
O3—S1—C1	107.6 (2)	O6—C14—C15	125.5 (5)
N1—S1—C1	106.8 (2)	N2—C14—C15	114.5 (4)
O4—S2—O5	119.9 (2)	O6—C14—N2	120.0 (5)
O4—S2—N2	102.2 (2)	C1—C2—H2A	120.00
O4—S2—C11	110.0 (2)	C3—C2—H2A	120.00
O5—S2—N2	108.8 (2)	C2—C3—H3	119.00
O5—S2—C11	108.0 (2)	C4—C3—H3	119.00
N2—S2—C11	107.2 (2)	C4—C5—H5	121.00
C4—O1—C7	119.0 (6)	C6—C5—H5	121.00
S1—N1—C8	128.4 (3)	C1—C6—H6	119.00
S2—N2—C14	124.4 (3)	C5—C6—H6	119.00
S1—N1—H1	116.00	O1—C7—H7A	109.00
C8—N1—H1	116.00	O1—C7—H7B	109.00
S2—N2—H2	118.00	O1—C7—H7C	109.00
C14—N2—H2	118.00	H7A—C7—H7B	110.00
S1—C1—C2	118.8 (5)	H7A—C7—H7C	110.00
S1—C1—C6	121.9 (4)	H7B—C7—H7C	110.00
C2—C1—C6	119.3 (6)	C8—C9—H9	120.00
C1—C2—C3	119.2 (6)	C10—C9—H9	120.00
C2—C3—C4	121.4 (6)	C9—C10—H10	121.00
O1—C4—C5	124.7 (7)	C11—C10—H10	121.00
C3—C4—C5	119.5 (7)	C11—C12—H12	120.00
O1—C4—C3	115.8 (6)	C13—C12—H12	120.00
C4—C5—C6	118.3 (6)	C8—C13—H13	120.00
C1—C6—C5	122.3 (6)	C12—C13—H13	120.00
C9—C8—C13	120.2 (4)	C14—C15—H15A	109.00
N1—C8—C9	116.8 (4)	C14—C15—H15B	109.00
N1—C8—C13	123.0 (4)	C14—C15—H15C	109.00
C8—C9—C10	120.5 (5)	H15A—C15—H15B	109.00
C9—C10—C11	118.8 (5)	H15A—C15—H15C	109.00
S2—C11—C10	119.4 (4)	H15B—C15—H15C	110.00
			
O2—S1—N1—C8	42.8 (5)	S2—N2—C14—O6	3.0 (7)
O3—S1—N1—C8	172.7 (4)	S2—N2—C14—C15	−175.4 (4)
C1—S1—N1—C8	−73.3 (5)	S1—C1—C2—C3	−178.1 (5)
O2—S1—C1—C2	171.0 (4)	C6—C1—C2—C3	−0.4 (8)
O2—S1—C1—C6	−6.7 (5)	S1—C1—C6—C5	178.2 (5)
O3—S1—C1—C2	40.0 (5)	C2—C1—C6—C5	0.6 (9)
O3—S1—C1—C6	−137.6 (5)	C1—C2—C3—C4	0.2 (9)
N1—S1—C1—C2	−72.1 (5)	C2—C3—C4—O1	−179.5 (6)
N1—S1—C1—C6	110.2 (5)	C2—C3—C4—C5	−0.2 (10)
O4—S2—N2—C14	176.6 (4)	O1—C4—C5—C6	179.6 (6)
O5—S2—N2—C14	48.9 (5)	C3—C4—C5—C6	0.3 (9)
C11—S2—N2—C14	−67.7 (4)	C4—C5—C6—C1	−0.5 (9)
O4—S2—C11—C10	30.0 (5)	N1—C8—C9—C10	178.9 (5)
O4—S2—C11—C12	−150.4 (4)	C13—C8—C9—C10	−0.7 (8)
O5—S2—C11—C10	162.5 (4)	N1—C8—C13—C12	−179.2 (4)
O5—S2—C11—C12	−17.9 (4)	C9—C8—C13—C12	0.4 (8)
N2—S2—C11—C10	−80.4 (4)	C8—C9—C10—C11	0.0 (8)
N2—S2—C11—C12	99.2 (4)	C9—C10—C11—S2	−179.4 (4)
C7—O1—C4—C3	178.1 (6)	C9—C10—C11—C12	1.1 (8)
C7—O1—C4—C5	−1.2 (9)	S2—C11—C12—C13	179.0 (4)
S1—N1—C8—C9	169.1 (4)	C10—C11—C12—C13	−1.4 (7)
S1—N1—C8—C13	−11.4 (7)	C11—C12—C13—C8	0.7 (8)

### Hydrogen-bond geometry (Å, °)

**Table:**

Cg1 and Cg2 are the centroids of the C1–C6 and C8–C13 rings, respectively.

**Table:**

*D*—H···*A*	*D*—H	H···*A*	*D*···*A*	*D*—H···*A*
N1—H1···O4^i^	0.86	2.09	2.932 (5)	168
N2—H2···O5^ii^	0.86	2.26	3.071 (5)	157
C13—H13···O2	0.93	2.35	2.986 (6)	126
C15—H15C···O6^ii^	0.96	2.45	3.348 (7)	156
C15—H15B···Cg1^iii^	0.96	2.79	3.722 (6)	164
C15—H15A···Cg2^iii^	0.96	2.79	3.589 (6)	141

Symmetry codes: (i) *x*, −*y*+1/2, *z*−1/2; (ii) *x*+1, *y*, *z*; (iii) −*x*+2, −*y*, −*z*+2.
